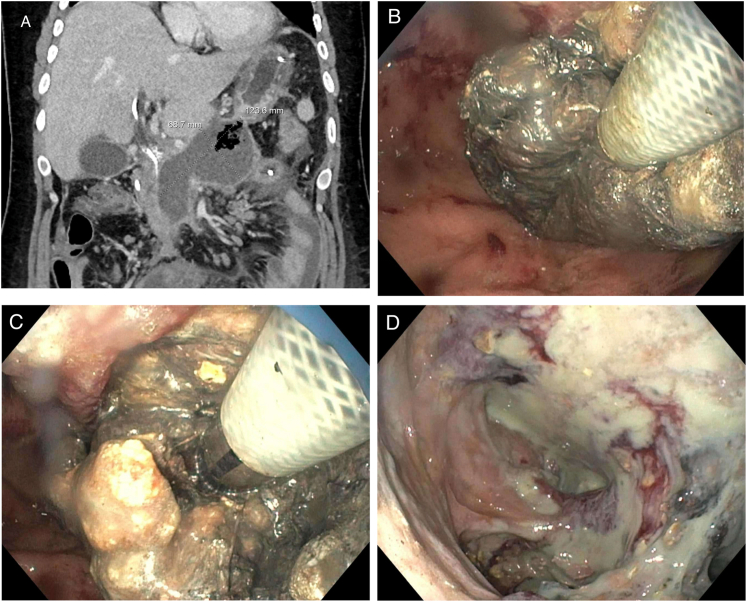# Novel Single-Session Intragastric Approach Necrosectomy Using a Large Over-the-Scope Endoscopic Morcellator

**DOI:** 10.1016/j.gastha.2026.100958

**Published:** 2026-04-07

**Authors:** Dylan Vainer, Drew Fuller, Christopher Ko

**Affiliations:** 1 Division of General Internal Medicine, University of Utah, Salt Lake City, Utah; 2 Division of Gastroenterology, Hepatology, and Nutrition, University of Utah, Salt Lake City, Utah

A 23-year-old man with a history of necrotizing gallstone pancreatitis complicated by a 12.4 × 6.9 cm walled-off necrosis status post cystogastrostomy 3 weeks prior presented with leukocytosis, tachycardia, and back pain concerning for infected necrosis (Figure A). Endoscopy revealed necrotic material protruding into the gastric lumen, and given favorable anatomy, a decision was made to proceed with intragastric necrosectomy using a large over-the-scope morcellator (EndoRotor, Interscope; Catheter 6.0-PED-EGD).

To improve exposure, the previously placed cystogastrostomy stent was removed. Necrosectomy was performed using a low rotational speed of 1000 rpm and a reduced vacuum setting of 300 mmHg. The suction side port (outer diameter 6.2 mm) was deliberately buried within necrotic tissue to ensure suction was transmitted exclusively to the necrosis while minimizing gastric decompression and mucosal collapse (Figure B and C). Using this targeted approach, necrotic tissue was gradually debrided, irrigated, and aspirated from the collection into the stomach, which was subsequently stented with 2 double-pigtail stents (Figure D). A complete necrosectomy was achieved in a single session entirely from the gastric lumen within 86 minutes. Despite known risks of stent dislocation, perforation, and bleeding, this case had no adverse events, highlighting the benefit of minimizing debridement sessions when clinically feasible.

While EndoRotor-assisted necrosectomy has been previously described, the novelty of this case lies in the intentional adjustment of suction and rotational parameters combined with precise port positioning to maintain focused debridement and avoid gastric collapse. Standard suction settings typically range from 500 to 620 mmHg; however, the reduced vacuum setting used here facilitated controlled tissue engagement while minimizing the risk to the surrounding gastric mucosa. This case highlights how favorable anatomy and device parameter modification can enable efficient, single-session necrosectomy in carefully selected patients while underscoring the importance of endoscopist experience and individualized procedural planning.

**Figure:**